# Inhibition of miR-383 suppresses oxidative stress and improves endothelial function by increasing sirtuin 1

**DOI:** 10.1590/1414-431X20198616

**Published:** 2020-01-24

**Authors:** Baoxiang Hu, Zushun Gong, Zhaohui Bi

**Affiliations:** Cardiac Intensive Care Unit, Zibo Central Hospital, Zibo, Shandong, China

**Keywords:** miR-383, Oxidative stress, Human umbilical vein endothelial cells, Sirtuin 1

## Abstract

Previous research has shown that suppression of miR-383 can prevent inflammation of the endothelium, as well as postpone the development of atherosclerosis. However, the role of miR-383 in endothelial cell apoptosis in diabetes remains unclear. The aim of this study was to investigate the function of miR-383 in high glucose-induced apoptosis and oxidative stress in endothelial cells. A series of experiments involving qualitative polymerase chain reaction, cell transfection, luciferase assay, assessment of cell death, detection of catalase and superoxide dismutase concentrations, detection of intracellular reactive oxygen species (ROS), and western blot analysis were performed in this study. We found that miR-383 expression was promoted, while NAD^+^-dependent deacetylase and sirtuin 1 (SIRT1) expressions were suppressed in the endothelium of the aorta in db/db mice as well as in human umbilical vein endothelial cells, which were treated with high glucose (HG). Increased expression of miR-383 decreased expression of SIRT1, while suppression of miR-383 promoted expression of SIRT1 in human umbilical vein endothelial cells (HUVECs). Furthermore, suppression of miR-383 following transfection with miR-383 suppressor repressed cell death and generation of ROS in HUVECs. SIRT1 knockdown by siRNA-SIRT1 reversed the suppressive effect of miR-383 inhibition on ROS production and cell apoptosis induced by HG treatment. Overall, the findings of our research suggested that suppression of miR-383 repressed oxidative stress and reinforced the activity of endothelial cells by upregulation of SIRT1 in db/db mice, and targeting miR-383 might be promising for effective treatment of diabetes.

## Introduction

Diabetes elevates the risk of cardiovascular diseases (CVD), including hypertension and atherosclerosis, due to endothelial dysfunction related to oxidative stress (OS) (1–3). In the diabetic state, excessive generation of reactive oxygen species (ROS) in the walls of vessels suppresses the bioavailability of nitric oxide (NO), decreasing endothelium-dependent relaxation and leading to severe cell injury and death (4,5). Consequently, methods to attenuate OS serve as potential ways to increase the activity of endothelial cells (ECs). Sirtuin 1 (SIRT1), an NAD+ (nicotinamide adenine dinucleotide)-dependent deacetylase, protects against oxidative stress and cell death (6,7). Emerging evidence indicates that microRNAs (miRNAs) modulate SIRT1 expression in various physiologic processes including stress resistance, apoptosis, and energy balance (8,9).

miRNAs, small endogenous non-coding RNAs, have been found to be biomarkers for various diseases (10). The function of miRNAs is modulated by binding to the 3′-UTR of target mRNAs, resulting in degeneration of mRNA and prohibition of translation (11). Previous studies have revealed that various miRNAs regulate the development of diabetes (12,13). Downregulation of miR-15a in the retina of patients with diabetes was found to counteract inflammation and generation of vessels (14). A study on mice with diabetes showed that the expression of miR-92a increased OS and inhibited the activity of ECs by binding to heme oxygenase-1 (15). miR-383 expression has been found to be suppressed before the onset of diabetes and to modulate death of pancreatic beta cells (16). miR-383 is also able to modulate cell death in various cancers (17,18). Expression of miR-383 triggers the death of breast cancer cells by promoting their sensitivity to DNA injury (19). Nevertheless, investigation of the expression patterns of miR-383 in the endothelium of patients with diabetes is insufficient.

In this study, we examined the expression of miR-383 in ECs in response to high glucose and determined the function of miR-383 in high glucose-induced apoptosis and oxidative stress in ECs. Furthermore, the target genes mediating the function of miR-383 in ECs were identified.

## Material and Methods

### Animals

C57BL/6 mice (glucose: 5±0.5 mmol/L, WT, n=4, male, 8 weeks old, Shanghai SLAC Laboratory Animal Co., Ltd., China) and db/db (Obese Leptin resistant mice suffering from type 2 diabetes) mice (glucose: 25±2.1 mmol/lL n=4, male, 8 weeks, Shanghai SLAC Laboratory Animal Co., Ltd.) were housed at the regulated conditions of 22–23°C, 55±5% humidity, and a 12-h light/dark cycle, with standard laboratory diet (Research Diet, Inc., USA) and water available *ad libitum*. All procedures related to animals complied with the Animal Experimentation Ethics Committee of Zibo Central Hospital.

### EC cultivation and high glucose supplementation

Human umbilical vein endothelial cells (HUVECs) (Lonza, USA) were cultivated in EC proliferation media (CC3024; Lonza) containing antibiotics and 10% FBS. Cells from passages 4 through 8 were used in further assays. As performed in previous studies, to prepare high glucose (HG) supplemented cell cultures, HUVECs were treated with D-glucose for 48 h, followed by investigation of miR-383 expression, cell death, and generation of ROS (20).

### Qualitative polymerase chain reaction (qPCR) of miR-383

Mice were anesthetized with an intraperitoneal injection of 350 mg chloral hydrate per kg animal body weight and decapitated. The thoracic aorta was quickly dissected out of the mouse chest, and the surrounding fat/connective tissue was removed in Krebs-Henseleit (KH) solution (mM: 118.0 NaCl, 2.52 CaCl_2_, 1.16 MgSO_4_, 24.88 NaHCO_3_, 1.18 K_2_PO_4_, 4.7 KCl, 10.0 glucose, 2.0 pyruvic acid, and 0.5 EDTA). Total RNA was isolated from HUVECs and aorta using TRIzol Reagent (Invitrogen, USA). RT-qPCR was carried out using Fast SYBR Green Master Mix (Applied Biosystems, USA) (21). The PCR cycling conditions were as follows: 5 min at 95°C, 36 cycles: 10 s at 95°C, 10 s at 58°C, and 20 s at 72°C. Normalization of concentration of miR-383 to U6 concentration was carried out. The sequence of the miR-383 primer was forward: 5′-GTGCAGGGTCCGAGGT-3, reverse: 5′-AGATCAGAAGGTGATTGTGGCT-3. The sequence of the U6 primer was forward: 5′-CTCGCTTCGGCAGCACA-3; reverse: 5′-AACGCTTCACGAATTTGCGT-3. The relative quantification of the target gene was conducted using the 2^-ΔΔCq^ method. U6 was used as the internal control for miRNA.

### Transfection of HUVECs with miR-383 suppressor

HUVECs were grown in 6-well plates at a density of 2×10^5^ cells/well for 24 h. The cells were then transfected with 80 nM miR-383 suppressor (5-AGCCACAAUCACCUUCUGAUCU-3, NC (negative control): 5-CAGUACUUUUGUGUAGUACAA-3, GenePharma Co., Ltd, China), or miR-383 mimic (5-AGAUCAGAAGGUGAUUGUGGCU-3, GenePharma Co., Ltd), or negative control (NC, 5-UUCUCCGAACGUGUCACGUTT-3) using Lipofectamine 2000 (Invitrogen) to knock down miR-383 expression. Cells proliferated in media without antibiotics for 24 h for use in additional assays. To assess knockdown of SIRT1, 40 nM of SIRT1 siRNA (GenePharma Co., Ltd) or control siRNA was transfected into HUVECs using Lipofectamine RNAiMAX (ThermoFisher, USA). siRNA and 10 μL of Lipofectamine RNAiMAX reagent (Invitrogen; Thermo Fisher Scientific, Inc.) was added to the culture medium and incubated for 6 h at 37°C. Subsequently, the transfection mixture was removed and cells were further incubated with normal medium for a further 24 h at 37°C. si-SIRT1 sense: 5′-CCGUCUCUGUGUCACAAAUTT-3′, si-SIRT1 anti-sense: 5′-AUUUGUGACACAGAGACGGTT-3′; control siRNA sense: 5′-UUCUCCGAACGUGUCACGUTT-3′, control siRNA anti-sense: 5′-ACGUGACACGUUCGGAGAATT-3′.

### Luciferase assay

HUVECs were co-transfected with pGL3-SIRT1, a luciferase plasmid containing SIRT1 3′UTR, and miR-383 or its suppressor using the Neon Transfection System (ThermoFisher). After 48 h, luciferase activities were measured with a Dual-Luciferase Reporter Assay System (Promega, USA). Firefly luciferase activity was normalized to Renilla luciferase activity.

### Assessment of cell death

HUVECs were collected and centrifuged at 1000 *g* for 5 min at 4°C for supernatant elution. Binding buffer was used to resuspend the pellet. FITC annexin V and propidium iodide (PI) were added prior to a 10-min incubation at room temperature. A FACScan flow cytometer (Becton, Dickinson and Company, USA) was used to examine fluorescent signals to assess cell death.

### Cell counting kit-8 (CCK-8) assay

The viability of the cells was assessed using the CCK-8 assay. In brief, at the predetermined time prior to the end of treatment, 100 μL of CCK-8 solution was added to each well, and the cells were incubated at 37°C or 4 h. The absorbance values at 450 nm were measured using a multi-well spectrophotometer (Bio-Rad, USA) at 450 nm.

### Detection of catalase (CAT) and superoxide dismutase (SOD1) activity

CAT and SOD1 activities were measured using a catalase assay kit and SOD assay kit (Cayman Chemical Co., USA) according to the manufacturer's instructions. The assay system consisted of 100 mM PBS (pH 7.0, 100 μL), methanol (30 μL) and sample (20 μL HUVEC from mouse aortas). The reaction was started by adding 35 μM H_2_O_2_ and the reaction mixture was incubated for 20 min at room temperature. After incubation, 10 M potassium hydroxide and chromogen were added to the mixture. After further incubation for 10 min, potassium periodate was added and incubated for 5 min at room temperature before reading the absorbance at 540 nm using a plate reader (Bio-Rad). CAT and SOD1 activities were calculated using the equation obtained from the linear regression of the standard curve.

### Detection of intracellular ROS

HUVECs were incubated for 30 min with 25 µmol fluorescent probe CM-H2DCFDA, then washed twice with PBS. To detect ROS inside cells, a multi-well fluorescent spectrophotometer was used at absorbance 485–530 nm. The intensity of generation of ROS in control group was manually set at 100%.

### Western blot analysis

HUVECs were homogenized using lysis buffer (Beyotime, China) and proteins were separated using 8–15% SDS-PAGE. The proteins were transferred to PVDF membranes (Millipore, USA), blocked for 1 h in 5% milk, and incubated with anti-β-actin and anti-SIRT1 (Cell Signaling Technology, USA) primary antibodies (mouse anti-SIRT1, 2028, 1:1000, Cell Signaling; mouse anti-β-actin, 3700, 1:5000, Cell Signaling) overnight at 4°C. Membranes were washed with PBS and then incubated with HRP-conjugated secondary antibodies (goat anti-mouse, 31430, 1:8000, ThermoFisher) for 1 h at 25°C. Enhanced chemiluminescence reagent (ThermoFisher Pierce) was applied for protein band detection. Beta-actin was used as a loading control for normalization in western blotting.

### Statistical analysis

Statistical analysis was performed by SPSS Statistics version 17.0 (USA) using ANOVA and Tukey's *post hoc* test. The results are reported as means±SE, with P<0.05 considered significant.

## Results

### miR-383 expression was promoted in diabetic murine aortas and in ECs exposed to HG

**Figure 1:**
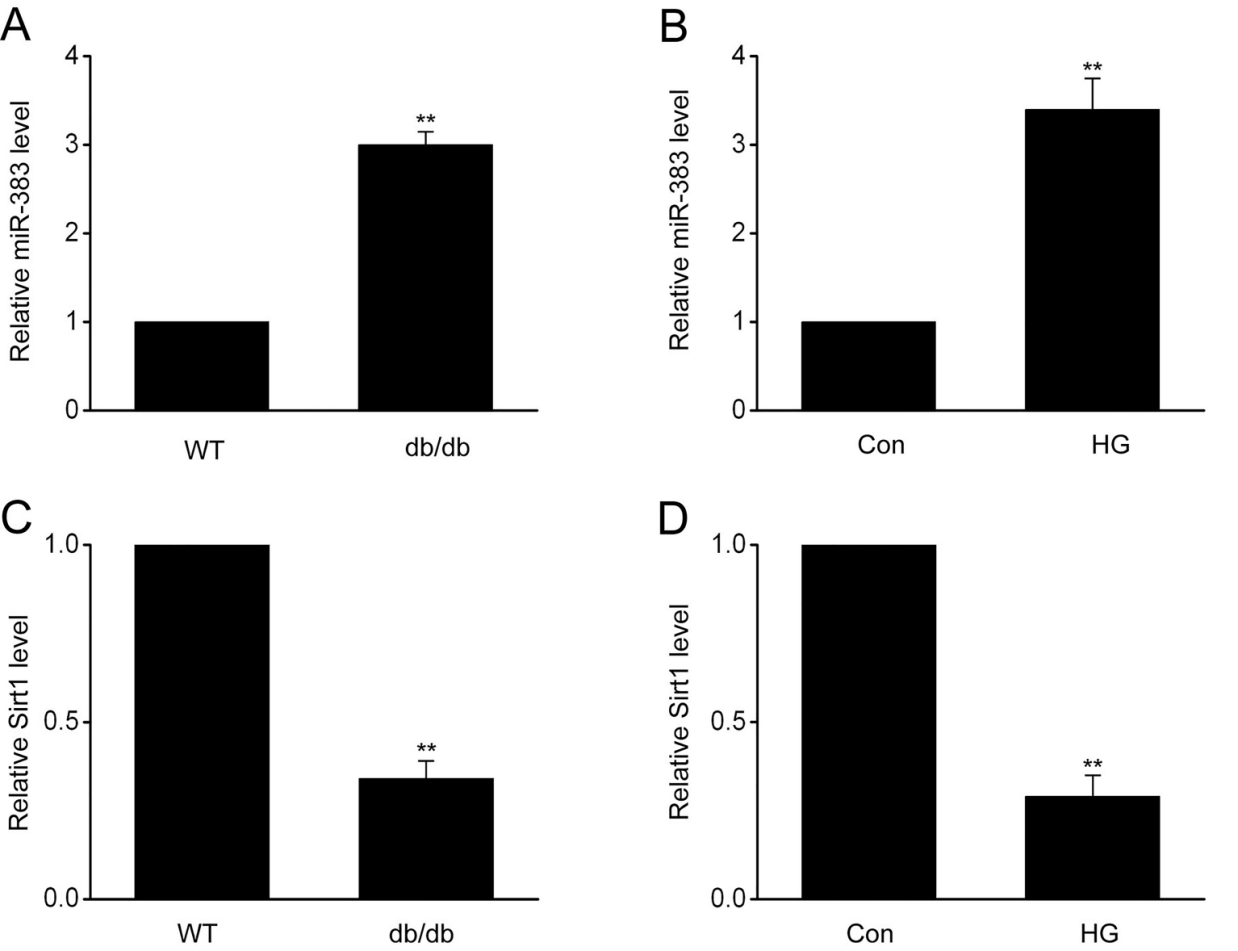
miR-383 expression was promoted in diabetic mice. Increased expression of miR-383 in (**A**) aortas of db/db mice and (**B**) human umbilical vein endothelial cells (HUVECs) supplemented with high glucose (HG) was evaluated by qRT-PCR. Knocked-down expression of SIRT1 in (**C**) aortas of db/db mice and (**D**) HUVECs supplemented with HG was evaluated by qRT-PCR. Data are reported as means±SE of 3 independent experiments. **P<0.01 (ANOVA and Tukey's *post hoc* test). WT: wild type; Con: control.

To examine the expression of miR-383 in diabetic mouse endothelial cell, RT-qPCR of miR-383 was carried out in db/db mice and HUVECs that received HG *in vitro*. As presented in Figure 1, miR-383 expression was increased in the aortas of mice with diabetes compared to wild type (WT) mice. Additionally, miR-383 expression was higher in HUVECs treated with HG than in control cells. Furthermore, expression of SIRT1 was suppressed in db/db mice and HUVECs treated with HG compared to controls. These findings indicated that increased expression of miR-383 may be related to the malfunction of ECs under diabetic conditions.

### miR-383 targeted SIRT1 in ECs

**Figure 2:**
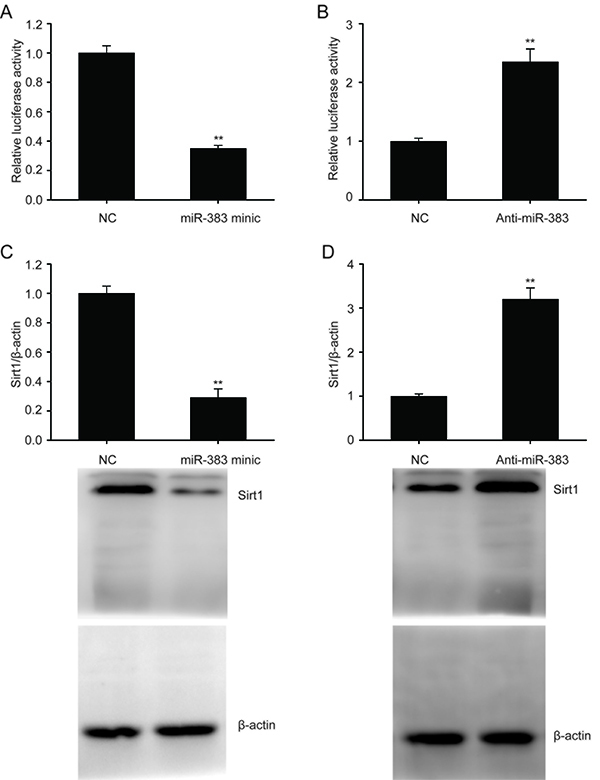
**A** and **B**, Luciferase function assay was used to examine the correlation between miR-383 and SIRT1. **C** and **D**, Representative immunoblots and quantitative evaluation of SIRT1 in human umbilical vein endothelial cells transfected with miR-383 mimic or miR-383 suppressor for 24 h. Data are reported as means±SE of 3 independent experiments. **P<0.01 (ANOVA and Tukey's *post hoc* test). NC: negative control.

The miRanda database (http://www.microrna.org) was used to filter and identify the putative target genes of miR-383. Bioinformatics analysis predicted that SIRT1 may serve as a target of miR-383. We found that increased expression of miR-383 suppressed the expression of SIRT1 in HUVECs and suppression of miR-383 promoted its expression (Figure 2A and B). Expression of SIRT1 was likely regulated by miR-383 binding directly to the 3′UTR and prohibiting translation. Suppression of miR-383 enhanced the expression of the SIRT1 protein in HUVECs (Figure 2C and D).

### Suppression of miR-383 decreased OS in ECs

**Figure 3:**
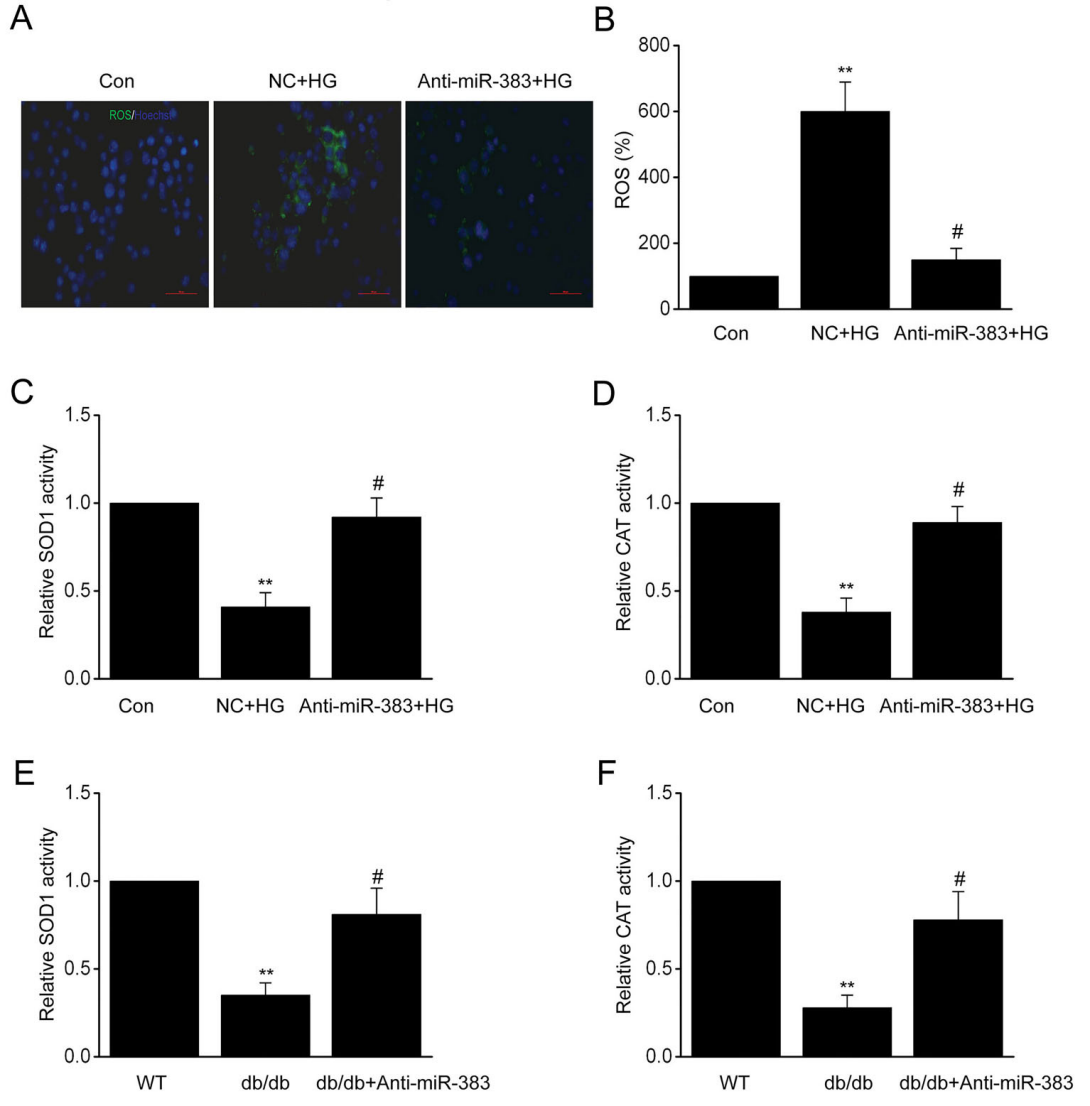
Human umbilical vein endothelial cells were transfected with miR-383 suppressor or negative control (NC) for 24 h before 48 h activation by treatment with high glucose (HG). **A**, Reactive oxygen species (ROS) inside the cells was assessed by the oxidation of H2DCFDA to DCF (magnification bars: 40 µm). **B**, Quantification of ROS. **C**, Activity of superoxide dismutase (SOD1) and **D**, catalase (CAT). **E**, Activity of SOD1 and **F**, CAT in aortas of db/db mice. Data are reported as mean±SE of 3 independent experiments. **P<0.01 *vs* control or WT; ^#^P<0.05 *vs* NC+HG group or db/db (ANOVA and Tukey's *post hoc* test). WT: wild type; Con: control.

It has been shown that increased OS contributes to the dysfunction of ECs under diabetic conditions (3). To investigate the influence of miR-383 on OS, we carried out loss of function assays by transfecting HUVECs with miR-383 suppressor or NC. Staining with CM-H2DCFDA fluorescent probe showed that ROS concentration increased in the HUVECs transfected with NC (Figure 3A and B). miR-383 suppression significantly repressed the generation of ROS in HUVECs. Furthermore, miR-383 suppression elevated CAT and SOD1 activity, as effective endogenous antioxidant, compared to the NC (Figure 3C and D). In addition, miR-383 suppression increased the activity of CAT and SOD1 in the aortas of db/db mice (Figure 3E and F). These findings proved that suppression of miR-383 represses excessive ROS generation in ECs under diabetic conditions.

### Suppression of miR-383 reduced cell death of ECs

**Figure 4:**
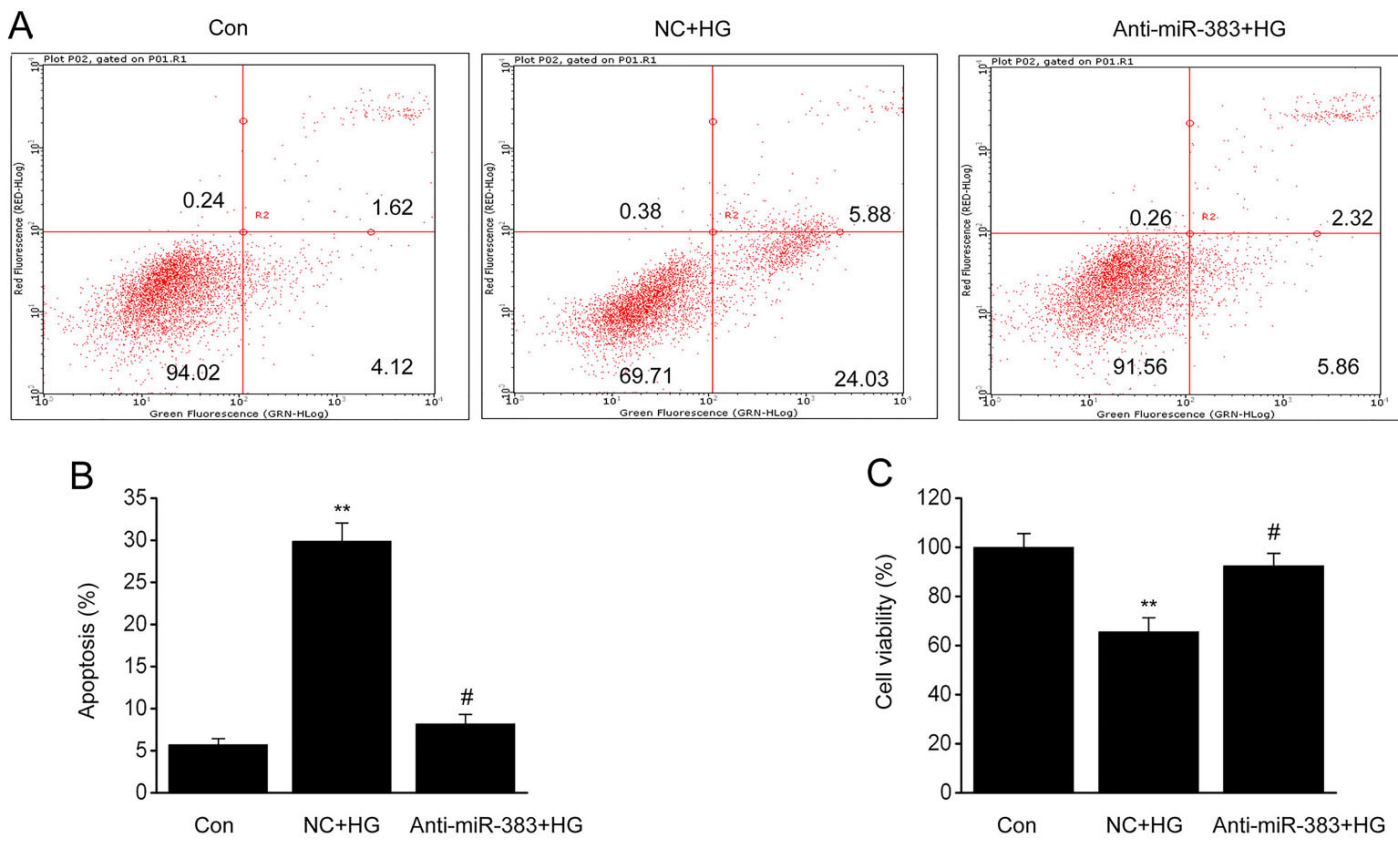
Human umbilical vein endothelial cells were transfected with miR-383 suppressor or negative control (NC) for 24 h prior to 48 h activation by treatment with high glucose (HG). **A**, Flow cytometry analysis of cell death. **B**, Quantification of cell death in each group. **C**, Cell viability was assessed by CCK-8 assay. Data are reported as means±SE of 3 independent experiments. **P<0.01 *vs* control; ^#^P<0.05 *vs* NC+HG group (ANOVA and Tukey's *post hoc* test). Con: control.

OS stimulation triggered by HG is an essential mechanism in ECs death (22). Thus, we examined the influence of miR-383 suppression on the death of HUVECs. Supplementation of the cells with HG increased the quantity of dead cells, which was counteracted by suppression of miR-383 (Figure 4A–C). Thus, targeting miR-383 could promote the viability of EC cells exposed to HG.

### SIRT1 knockdown counteracted the influence of miR-383 suppression on ROS generation and ECs death

**Figure 5:**
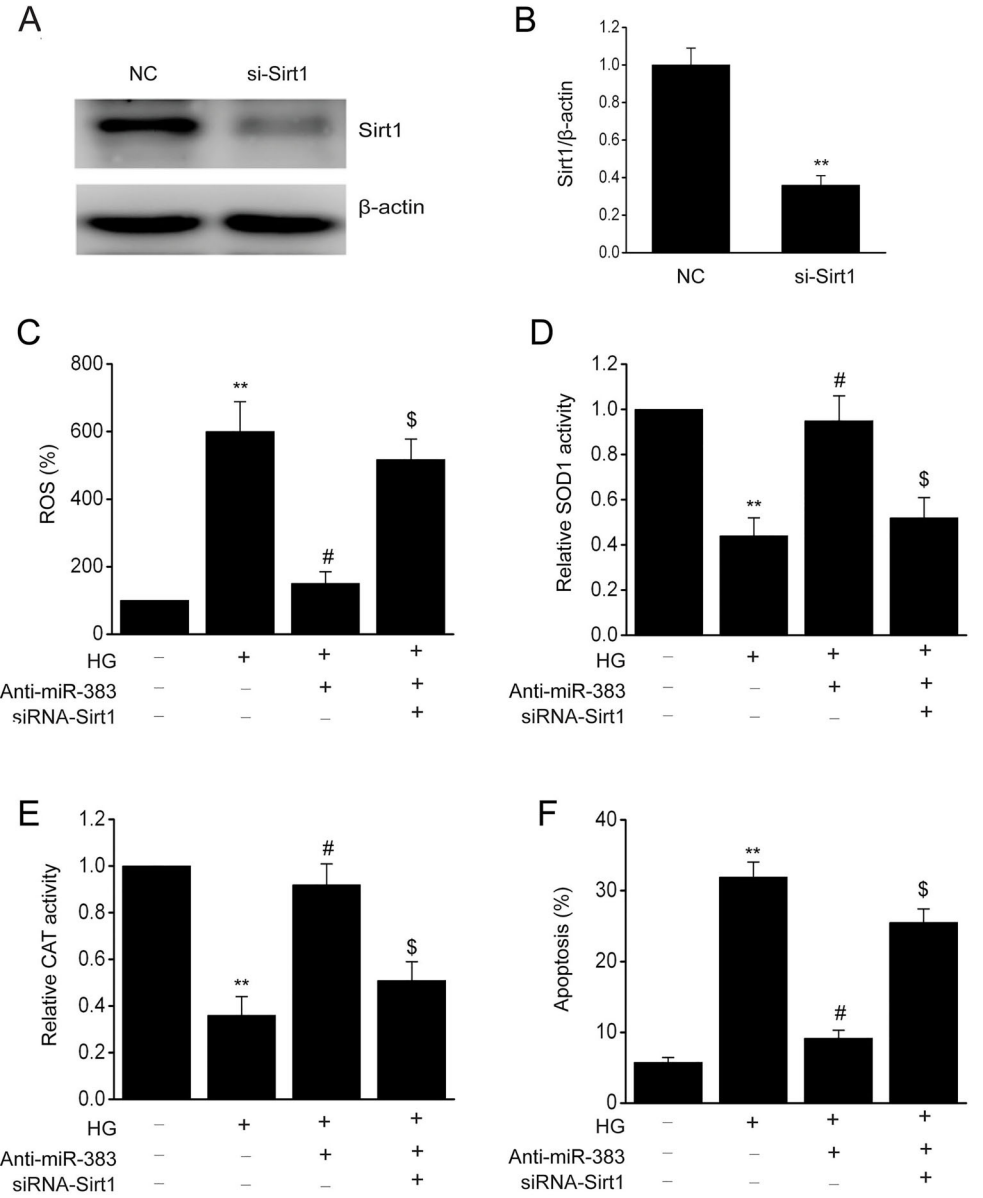
Human umbilical vein endothelial cells (HUVECs) were transfected with SIRT1 siRNA and miR-383 suppressor for 24 h prior to 48 h activation by treatment with high glucose (HG). **A** and **B**, Representative immunoblot and quantitative evaluation of SIRT1 in HUVECs. **C**, Reactive oxygen species (ROS) inside the cells was assessed by oxidation of H2DCFDA to DCF. **D**, Activity of superoxide dismutase (SOD1) and **E**, catalase (CAT) in groups. **F**, Flow cytometry analysis of cell death. Data are reported as means±SE of 3 independent experiments. **P<0.01 *vs* control; ^#^P<0.05 *vs* negative control (NC)+HG group; ^$^P<0.05 *vs* anti-miR-383 group (ANOVA and Tukey's *post hoc* test).

To examine if SIRT1 regulated the influence of miR-383 suppression on the attenuation of OS and promotion of EC viability, siRNA was used to knock down expression of SIRT1 in HUVECs (Figure 5A and B). The influence of miR-383 suppression on the ROS generation by HUVECs following exposure to HG was counteracted by knockdown of SIRT1. Knockdown of SIRT1 repressed the reinforcement of CAT and SOD1 that is usually triggered by suppression of miR-383. Furthermore, the inhibitory influence of miR-383 suppression on HG-triggered death of HUVECs was counteracted by knockdown of SIRT1 (Figure 5C–F). These results showed that suppression of ROS generation and death of ECs by miR-383 can be modulated by knockdown of SIRT1.

## Discussion

miRNAs are essential contributors to the modulation of CVD by targeting various genes (23,24). miR-383 is involved in various cellular processes related to the development of various diseases, such as generation of malignancies and cerebral ischemia (25–27). Our study found an increased expression of miR-383 in murine aortic ECs from db/db mice that were treated with HG. It was revealed that increased expression of miR-383 in diabetic murine ECs reinforced OS, and suppression of miR-383 impaired cell death and normalized ROS generation in ECs under diabetic conditions.

miR-383 targeted SIRT1, whose upregulation promoted endothelial activity by suppressing miR-383 expression in db/db mice. These findings emphasized the promising influence of miR-383 on regulation of malfunctioning vessels in patients suffering from diabetes. Targeting of miR-383 may be a way to treat vessel pathologies in diabetes.

Excessive ROS generation contributes to EC malfunction in diabetes (28). Previous studies have shown that ROS generation partly contributes to HG-triggered cell death of ECs (29,30). OS normalization, resulting from use of antioxidants, reduces damage to vessels in patients with diabetes (31,32). We found that suppression of miR-383 reduced ROS generation, elevated the levels SOD1/CAT, and repressed ECs death that were supplemented with HG. This indicated that endothelial activity was promoted through miR-383 suppression in ECs by prohibition of ROS under diabetic conditions.

Our study revealed the target gene that could participate in preserving the influence of miR-383 suppression on ECs in patients with diabetes. We screened for enzyme expression related to OS in HUVECs transfected with miR-383 suppressor and found promotion of SIRT1 translation. We confirmed that suppression of miR-383 directly promoted expression of SIRT1 in ECs. Although previous research has explored the etiology of SIRT1 modulation in ECs, it has not been well studied in diabetes (33,34). In the present study, we found that SIRT1 was downregulated in HUVECs treated with HG. Downregulation of SIRT1 was also seen in ECs from the aortas of db/db mice. Our research has shown that SIRT1 suppressed miR-383 expression in diabetic murine ECs to preserve their influence on vessels.

In summary, our study provided evidence that targeting miR-383/SIRT1 axis can be an alternative therapeutic strategy for ameliorating diabetic vascular degeneration.
